# The role of community pharmacists in immunisation: a national cross-sectional study

**DOI:** 10.1007/s11096-021-01357-5

**Published:** 2021-11-26

**Authors:** Nikolaus Lindner, Martin Riesenhuber, Thomas Müller-Uri, Anita Elaine Weidmann

**Affiliations:** 1 School of Pharmacy and Life Sciences, Robert Gordon University, Garthdee Road, Aberdeen, AB10 7QB Scotland, UK; 2 Division of Cardiology, Department of Internal Medicine II, Medical University of Vienna, Vienna, Austria; 3 Vindobona-Apotheke, Community Pharmacy, Vienna, Austria; 4 Faculty of Chemistry and Pharmacy, University of Innsbruck, Innsbruck, Austria

**Keywords:** Community pharmacy, Covid, Pharmacist immunisation, Questionnaire, Service implementation, Survey, Vaccine

## Abstract

*Background* Austrian pharmacists are not authorised to administer immunisations, and evidence about their willingness to immunise is lacking. *Aim* The aim of this study is to investigate Austrian community pharmacists’ willingness to administer immunisations in the future. *Method* This study is designed as a cross-sectional online survey based on the theoretical domains framework (TDF). The validated and piloted questionnaire obtained ethical approval by Robert Gordon University. Outcome measures included pharmacists’ willingness to immunise, service requirements, barriers and education needs. *Results* The questionnaire was sent out to 3086 community pharmacists of which 380 responses were included in the final analysis (12.3%). Willingness to administer immunisations after appropriate training and legislative regulation was stated by 82.6% (*n* = 314) of participants. It was demonstrated that pharmacists willing to immunise were significantly younger than their counterpart (38 [IQR 31–49] years vs. 45 [IQR 37.5–54] years; OR 1.06; 1.03–1.09, 95% CI; *p* < 0.001). ‘Legal liability’ was considered the most critical barrier to service implementation, ‘seeing blood’ and ‘close patient contact’ as least critical. Pharmacists not willing to immunise showed a higher probability to evaluate personnel resources (OR 2.98; 1.35–6.58, 95% CI; *p* = 0.007), close patient contact (OR 2.79; 1.46–5.34, 95% CI; *p* = 0.002) and management of side effects (OR 2.62; 1.21–5.67, 95% CI; *p* = 0.015) as (highly) critical. The majority assessed the ‘right timing for training’ to be after the foundation training with a 2-yearly renewal. *Conclusion* Austrian community pharmacists show a strong willingness to administer immunisations while highlighting important requirements and barriers towards service implementation.

## Impact on Practice

- Austrian community pharmacists are not authorised to immunise, but report a high willingness to administer vaccines in the future.
- This study serves as a valuable instrument for stakeholders attempting to implement a pharmacist provided immunisation-service.
- Legalisation of vaccine administration will enable pharmacists to take on a patient-centred role in the Austrian health care system.
- Pharmacist provided immunisation-services will facilitate access to vaccines for patients.

## Introduction

According to the World Health Organisation (WHO), immunisations in general prevent 2–3 million deaths each year across all age groups [1]. Vaccines themselves represent a successful and cost-effective measure to combat numerous infectious diseases of varying severity. To improve access to vaccinations, many countries have already expanded the role of pharmacists from immunisation educators to active immunisers in the community setting [2]. Alongside big healthcare systems such as Canada, USA and Australia, several European countries such as the United Kingdom, France and Portugal have introduced pharmacist-provided immunisation services [3]. A meta-analysis from Baroy et al. investigated pharmacist-provided vaccination services in the USA in 5 community sites and 3 hospitals including 11 study arms [4]. An increase in overall immunisation rates delivered by pharmacists’ immunisation programmes was shown compared to usual care (RR = 2.95, *p* < 0.001) and varied based on the type of vaccine with the highest risk ratio for the herpes vaccine subgroup (RR = 4.78, *p* < 0.001), and the smallest for the flu vaccine subgroup (RR = 2.23, *p* < 0.001). Isenor et al. performed a pooled analysis of two randomised controlled trials examining pharmacist-provided immunisation services in the USA [5]. This analysis demonstrated a significant increase in overall immunisation rates compared to normal immunisation service provision (RR = 2.64, 1.81–3.86, 95% CI). A more recent systematic review by Spinks et al. from 2020 further suggests that pharmacist-provided vaccinations may additionally result in reduced costs and improved convenience [6]. While these settings are not directly comparable to the Austrian health care system, the Austrian Chamber of Pharmacists maintains that community pharmacists remain the most easily accessible health care professional with 95% of the Austrian public able to reach a community pharmacy within 10 min [7]. Up to now, Austrian community pharmacists’ willingness towards administering vaccines remains unexplored. In Austria, only physicians and nurses under the supervision of a physician are authorised by law to administer immunisations. The threat of the emerging Covid-19 pandemic and expected low flu immunisation rates resulted in a proposal of a pharmacist-provided immunisation service in early 2020. While this aimed at providing a valuable strategy to improve access to vaccination services across the general public, it has caused controversial discussions among stakeholders. Austrian community pharmacists are advocating for the introduction of pharmacist-provided immunisation services not only to accelerate Covid-19 vaccination coverage, but also to combat low flu immunisation rates and increasing tick borne encephalitis cases [8, 9]. However, proposals are confronted with safety concerns by medical stakeholders and opposing political parties [10, 11].

In light of the ongoing Covid-19 pandemic it is essential to quickly increase immunisation uptake to possibly attain herd immunity and prevent further disease outbreaks, while also maintaining general and flu vaccination coverage. A potent strategy to increase vaccination uptake is to improve access to vaccination services [12].

### Aim

The aim of this study is to investigate Austrian community pharmacists’ willingness to administer immunisations in the future.

### Ethics approval

Ethical approval for the study was obtained by the School Research Ethics Committee of Robert Gordon University, Aberdeen on the 7th December 2020. The Ethics Committee of the City of Vienna advised that no further ethical approval was needed.

## Method

### Study design and data collection tool development

This study is a cross-sectional online survey that was designed using Jisc ‘online surveys’ (https://www.onlinesurveys.ac.uk/). Primary outcomes included the percentage of employed Austrian community pharmacists willing to administer immunisations, a ranking of relevant requirements and barriers to implementation of an immunisation service and desired training specifications. Secondary outcomes included matters brought up in the open-response section of the questionnaire. The questionnaire was based on best practice guidelines and the Theoretical Domains Framework (TDF), an integrative framework validated as a method ‘for theoretically assessing implementation problems’ [13–15]. The following domains of the TDF were addressed in the study: skills, professional role and identity (section 1); beliefs about capabilities, intentions, goals, environmental context and resources, social influences (section 2); goals (section 3). Answer options consisted of open/closed questions, Likert-scale and semantic differential questions.

Participating pharmacists were provided assurance of anonymity in the invitation E-mail, the information leaflet and at the beginning of the questionnaire in line with the British Psychological Society’s Ethics Guidelines for Internet-mediated Research [16]. The survey with all corresponding materials (information leaflet, email covering note) was pretested for face and content validity among five research experienced pharmacy practitioners. The questionnaire was adapted according to feedback on survey design and content appropriateness. Piloting was carried out in 36 Austrian pharmacists (10% of final sample size). Feedback from 21 responding participants (response rate: 58.3%) as well as technical issues and trends in (non)-responses were analysed and the questionnaire was modified accordingly. Responses of the pilot were not included in the final analysis.

### Study population and sample size

A sample size calculation was performed based on all registered employed community pharmacists in Austria (*n* = 4575). A margin of error of 5% and a confidence level of 95% were considered as adequate for statistical analysis. This resulted in a sample size of 355 pharmacists. The questionnaire was distributed by the Austrian Association of Employed Pharmacists (VAAÖ) and Forum Pharmazie Vienna to all its members for whom email addresses were available (*n* = 3086). Pharmacists who were retired, non-German speaking or not working in community pharmacy were excluded. One reminder email was sent after one week. Data was collected over a period of 5 weeks between 19th January 2021 and 23rd February 2021.

### Data analysis

Statistical analysis was performed using SPSS (vs 21.0) and included descriptive statistics, cross-tabulations, relevant parametric/non-parametric tests and regression analyses. Continuous variables were tested for normal distribution by Kolmogorov–Smirnov test. For normal distributed continuous variables, means ± standard deviations were calculated. For non-normal distributed continuous variables, medians ± interquartile ranges (IQR) were calculated. Groups with continuous variables were compared with the t-test (normal distribution) or with the Mann–Whitney U test (non-normal distribution). Discrete variables were compared with the chi-square test. Odds ratios were calculated with a logistic regression model, and 95% confidence intervals were reported. Parameters were included in the multifactorial regression if the p value was < 0.1 in the univariate regression model. A two-sided *p* value < 0.05 was considered statistically significant.

## Results

### Response rate

Out of 433 respondents, 46 (10.6%) were excluded for being hospital pharmacists, and 7 (1.6%) were excluded for working in other fields of practice. In total, 380 community pharmacists (87.8%) were included in the final analysis, which results in a response rate of 12.3%. This represents 8.3% of all employed community pharmacists (*n* = 4575) in Austria and meets the calculated sample size expectation [7]. Previous studies distributed to Austrian community pharmacists reported response rates of approximately 3–19.1% [17, 18].

### Socio-demographic characteristics

Detailed participant characteristics stratified by willingness to immunise are displayed in Table 1. Out of a total sample of 380 participants, 341 (89.7%) were female. This is representative of the demographics of community pharmacists in Austria, where 86.7% (*n* = 3967) of all employed community pharmacists are female [7]. Participants’ median age was 40 [IQR 32–50] years with a median work experience of 12 [IQR 4–22] years. The majority of participants were pharmacists that worked in pharmacies with 5–10 employees (*n* = 162; 42.6%) or in pharmacies with 11–20 employees (*n* = 172; 45.3%).

**Table 1:** Collective participant characteristics stratified by willingness to immunise

	Total	Willing to immunise	Not willing to immunise	*p* value
Total sample—*n (%)*	380 (100.0%)	314 (82.6%)	66 (17.4%)	
Age (years) – *median [IQR]*	40 [32–50]	38 [31–49]	45 [37.5–54]	< 0.001*
Work experience (years) – *median [IQR]*	12 [4–22]	10 [4–20]	20 [10.5–25]	< 0.001*
*Sex*—*n (%)*				
Female	341 (89.7%)	280 (89.2%)	61 (92.4%)	0.048**
Male	38 (10.0%)	34 (10.8%)	4 (6.1%)	
Not specified	1 (0.3%)	0 (0.0%)	1 (1.5%)	
*Level of education*—*n (%)*				
Mag. pharm.	350 (92.1%)	291 (92.7%)	59 (89.4%)	0.706**
Dr./PhD	26 (6.8%)	20 (6.4%)	6 (9.1%)	
Other	1 (0.3%)	1 (0.3%)	0 (0.0%)	
Not specified	3 (0.8%)	2 (0.6%)	1 (1.5%)	
*Postgraduate degree*—*n (%)*				
Yes	56 (14.7%)	48 (15.3%)	8 (12.1%)	0.218*
No	309 (81.3%)	256 (81.5%)	53 (80.3%)	
Not specified	15 (3.9%)	10 (3.2%)	5 (7.6%)	
*Immunisation counselling*—*n (%)*				
Yes	364 (95.8%)	303 (96.5%)	61 (92.4%)	0.134*
No	16 (4.2%)	11 (3.5%)	5 (7.6%)	
*Workplace (employees)*—*n (%)*				
< 5 employees	9 (2.4%)	7 (2.2%)	2 (3.0%)	0.003*
5–10 employees	162 (42.6%)	130 (41.4%)	32 (48.5%)	
11–20 employees	172 (45.3%)	148 (47.1%)	24 (36.4%)	
> 20 employees	32 (8.4%)	28 (8.9%)	4 (6.1%)	
Not specified	5 (1.3%)	1 (0.3%)	4 (6.1%)	
*Workplace (inhabitants)*—*n (%)*				
< 1.000 inhabitants	2 (0.5%)	1 (0.3%)	1 (1.5%)	0.031*
1.000–9.999 inhabitants	135 (35.5%)	106 (33.8%)	29 (43.9%)	
10.000–49.999 inhabitants	56 (14.7%)	49 (15.6%)	7 (10.6%)	
50.000–99.999 inhabitants	15 (3.9%)	15 (4.8%)	0 (0.0%)	
100.000–499.999 inhabitants	44 (11.6%)	37 (11.8%)	7 (10.6%)	
> 500.000 inhabitants	123 (32.4%)	104 (33.1%)	19 (28.8%)	
Not specified	5 (1.3%)	2 (0.6%)	3 (4.5%)	

*Mann–Whitney U test, **Chi-square test

### Willingness to immunise

In total, 314 of 380 community pharmacists (82.6%) expressed willingness to administer immunisations after appropriate training and legislative regulation (Table 1). Regression analysis showed that pharmacists willing to immunise were significantly younger than pharmacists not willing to immunise (38 [IQR 31–49] years vs. 45 [IQR 37.5–54] years; OR 1.06, 95% CI 1.03–1.09, p < 0.001). No statistically significant difference was seen across genders. In relation to patient groups that pharmacists would administer vaccines to, all pharmacists are willing to immunise adults from 18 to 65 years (*n* = 314, 100.0%), whereas only 15.6% (*n* = 49) of participants would administer vaccines to children under 14 years of age (Table 2). Vaccine types that pharmacists are willing to administer are displayed in Table 3.

**Table 2:** Patient groups pharmacists are willing to immunise (*n* = 314)

Patient groups	*n*	%
Adults (18–65 years)	314	100.0
Elderly people (over 65 years)	239	76.1
Adolescents (14–17 years)	174	55.4
Children (under 14 years)	49	15.6
Other	4	1.3

**Table 3:** Vaccine types pharmacists are willing to administer (*n* = 314)

Vaccine types	*n*	%
Influenza	302	96.2
Tick-borne encephalitis	298	94.9
General vaccinations	259	82.5
Covid-19	206	65.6
Travel vaccinations	161	51.3
Other	14	4.5

### Requirements for service implementation

The majority of participants regarded ‘appropriate training’ (*n* = 336, 88.4%), ‘liability insurance’ (*n* = 297, 78.2%) and ‘acceptance by patients’ (*n* = 280, 73.7%) as highly important (Table 4). ‘Acceptance by physicians’ and ‘financial remuneration’ were considered as less important, with only 39.2% (*n* = 149) and 30.8% (*n* = 117) of participants rating these aspects as highly important. In the open comments, participants added that further important requirements include ‘adequate pharmacy premises’, ‘acceptance by politics and stakeholders’, ‘legal implementation’ and ‘appropriate training incorporating first-aid measures’. One pharmacist pointed out that ‘*future legal regulation and solid education will act as proof that pharmacists are equally qualified to vaccinate compared to other healthcare professionals*’ [P-71247183].

**Table 4:** Requirements for service implementation (*n* = 380)

Topic	Highly important	Important	Hardly important	Not important
Appropriate training	88.4% (336)	11.3% (43)	0.3% (1)	0.0% (0)
Liability insurance	78.2% (297)	20.3% (77)	1.6% (6)	0.0% (0)
Acceptance by patients	73.7% (280)	25.3% (96)	0.8% (3)	0.3% (1)
Acceptance by physicians	39.2% (149)	39.2% (149)	18.2% (69)	3.4% (13)
Financial remuneration	30.8% (117)	49.7% (189)	18.4% (70)	1.1% (4)

Data is displayed as % (*n*)

### Barriers to the administration of immunisations

**Fig. 1:**
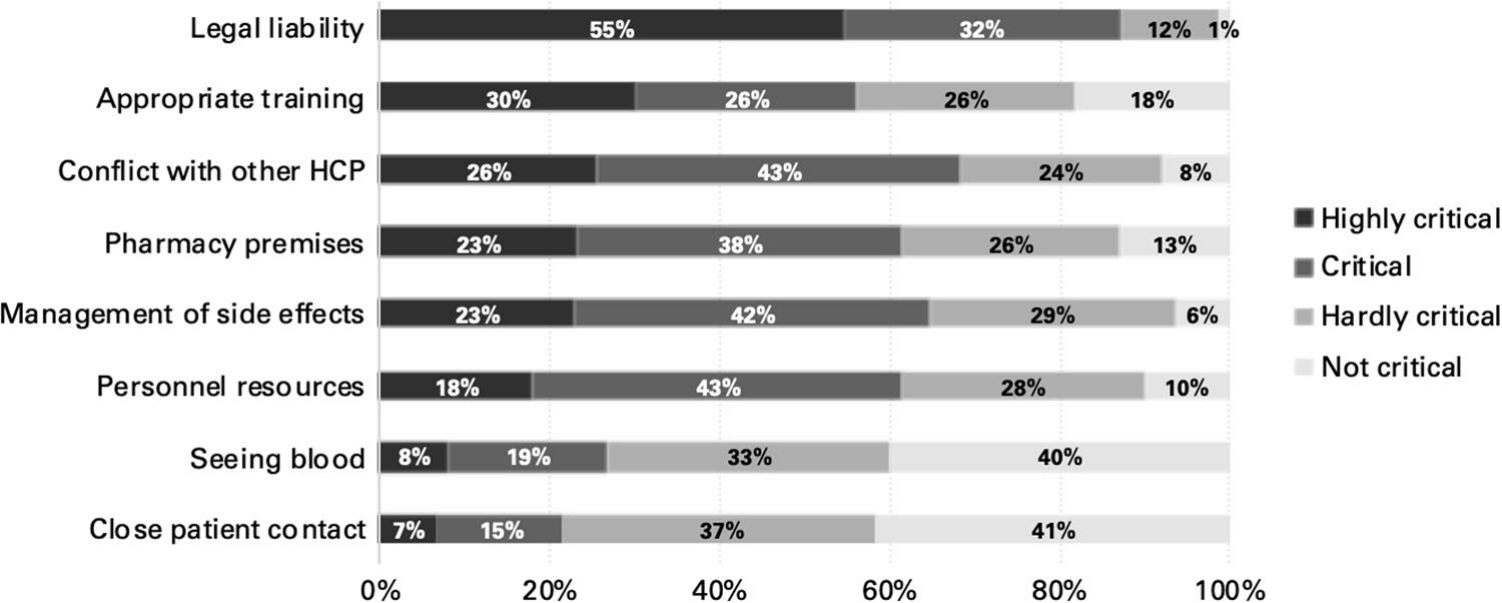
Pharmacist reported barriers for the administration of immunisations (*n* = 380)

‘Legal liability’ was considered the most important barrier with 54.7% (*n* = 208) of participants rating this aspect as highly critical (Fig. 1). In addition, more than 50% of the participants regarded the following barriers as highly critical and critical: ‘conflict with other healthcare professionals’ (*n* = 260, 68.4%), ‘management of side effects’ (*n* = 246, 64.8%), ‘adequate pharmacy premises’ (*n* = 234, 61.6%), ‘personnel resources’ (*n* = 234, 61.6%) and ‘appropriate training’ (*n* = 214, 56.4%). The minority of participants rated the ‘sight of blood’ (*n* = 102, 26.9%) and ‘close patient contact’ (*n* = 83, 21.8%) as highly critical and critical.

**Fig. 2:**
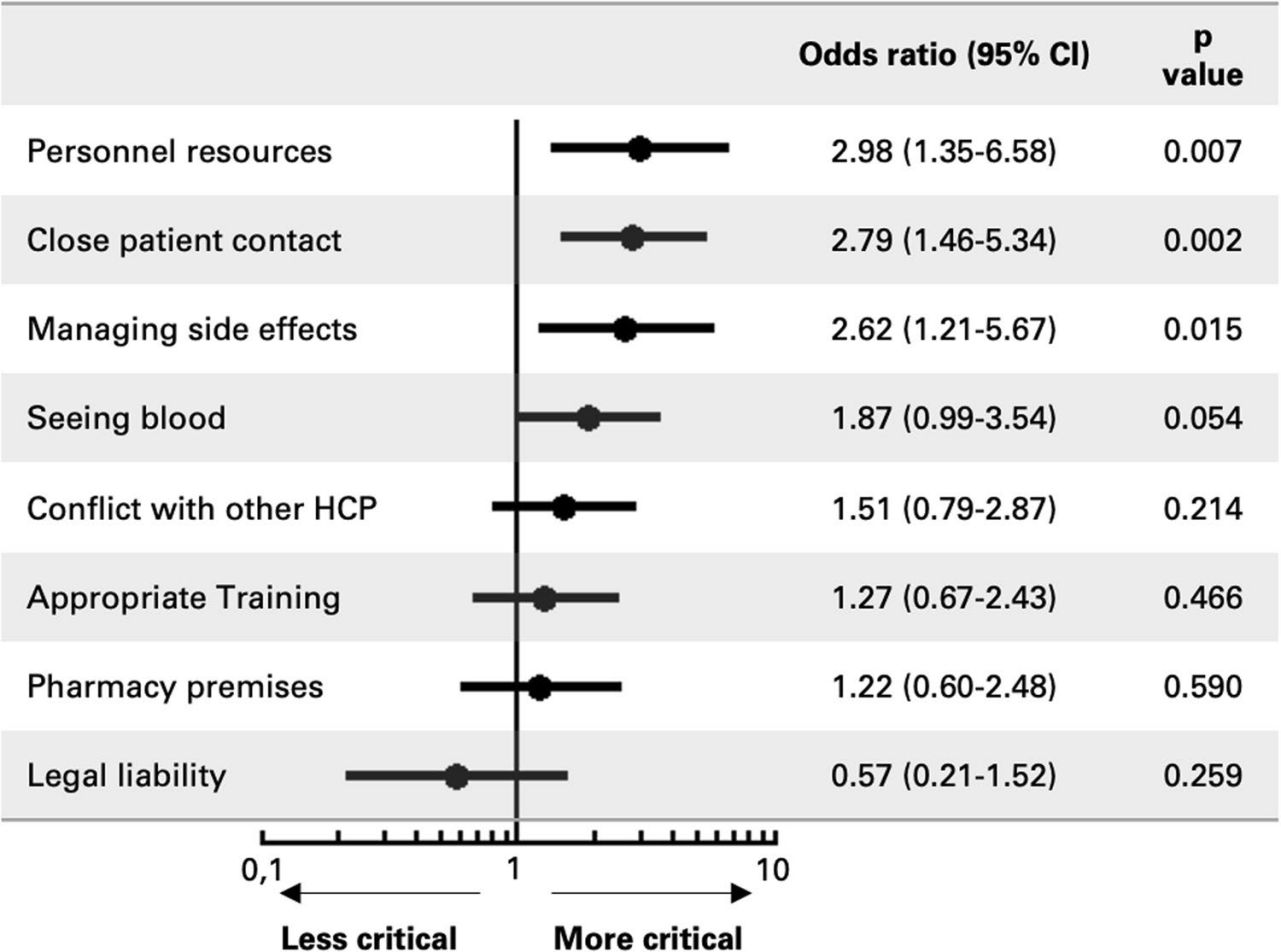
Critical barriers identified by pharmacists not willing to administer immunisations

In comparison to pharmacists willing to immunise, pharmacists not willing to immunise more probably rated ‘personnel resources’ (OR 2.98, 95% CI 1.35–6.58, p = 0.007), ‘close patient contact’ (OR 2.79, 95% CI 1.46–5.34, *p* = 0.002) and ‘management of side effects’ (OR 2.62, 95% CI 1.21–5.67, *p* = 0.015) as critical/highly critical (Fig. 2). Participants that work in a pharmacy with ≤ 10 employees more probably rated ‘management of side effects’ (OR 1.58, 95% CI 1.03–2.43, *p* = 0.037) and ‘personnel resources’ (OR 1.55, 95% CI 1.02–2.36, *p* = 0.042) as critical/highly critical in comparison to participants that work in a pharmacy with more than 10 employees. In contrast to these findings, participants that work in a pharmacy with ≤ 10 employees less probably rated ‘conflict with other HCP’ as critical/highly critical (OR 0.65, 95% CI 0.42–1.00, *p* = 0.052), albeit not statistically significant.

Additional barriers identified in the free text responses included ‘fear of needles’, ‘acceptance by patients/physicians’, ‘management of emergencies/side effects’ and ‘delineation of job profiles for physician and pharmacist’. One pharmacist added that ‘*physicians and their stakeholders represent an insurmountable hurdle in Austria’ [P-70820348]*. Another pharmacist pointed out that they *‘do not consider administering vaccinations as a pharmaceutical task’ [P-70823562]*.

### Education and training

The majority of participants prefer training to be held after the foundation training (*n* = 173, 45.5%) with a 2-yearly renewal interval (*n* = 136, 35.8%). Almost all participants regarded ‘first aid’ (*n* = 347, 91.3%), ‘assessment of indications and contraindications’ (*n* = 345, 90.8%) as well as ‘practical administration’ (*n* = 342, 90.0%) as highly relevant topics for the immunisation training programme. Topics such as the ‘vaccination schedule’, ‘preventable diseases’, ‘travel vaccinations’ and ‘(bio)chemical characteristics’ were considered as less relevant. Other important topics specified in the open response options included ‘handling of special patient groups’, ‘communication’, ‘legal basis’, ‘logistics’, ‘hygiene and documentation’. One pharmacist added that ‘*besides educational documentation it is vital to document and evaluate vaccination uptake after introduction of the pharmacist-provided service*’ *[P-70862523]*. Another pharmacist emphasised that *‘the communicational aspect of interacting with vaccination sceptics should be included in the training’ [P-70850050].*

## Discussion

The results presented in this study clearly demonstrate that community pharmacists in Austria show a strong willingness to administer immunisations with 82.6% (*n* = 314) of respondents being in favour. However, appropriate training, liability insurance and acceptance by patients were considered to be highly important requirements for the successful implementation of a pharmacist-provided immunisation service. This study is the first to explicitly investigate Austrian community pharmacists’ willingness to deliver a vaccination service in the future. Despite the reasonably low response rate of 12.3% (*n* = 380), the study meets its sample size target for the pharmacist population across all of Austria. The gender distribution of study participants is comparable to the latest community pharmacy census study [7]. Major limitations of the study lie in its nature of a questionnaire study and may have resulted in a potential (non-) response and selection bias. The questionnaire was not distributed to the whole population of 4575 Austrian employed community pharmacists, however to a sample of 3086 pharmacists (67.5%). Reasons for this restricted distribution were institutional policies by the Austrian Chamber of Pharmacists. This limitation may lead to a potential selection bias. Moreover, it cannot be excluded that the questionnaire was distributed to other pharmacists that are not members of the distributing associations. However, through the survey design it was assured that only non-retired, German speaking community pharmacists have been selected for the final analysis. Another limitation is the sole representation of the pharmacists’ perspective. The attitude of other healthcare professionals, patients and policy makers towards implementation of a pharmacist-provided immunisation service should be addressed in further work as they can act as limiting factors upon and after implementation of such a service.

In 2015, Edwards et al. also reported an overwhelming willingness of community pharmacists in Canada (68.0%, *n* = 337) to administer immunisations [19]. However, a high number of respondents (43.0%, *n* = 213) were pharmacy owners or managers. Their attitudes can differ from those of employed community pharmacists possibly due to their age and economic orientation. Moreover, a potential response bias may have been incurred in our study as a consequence of the current national debate around pharmacists’ ability to immunise in Austria. These circumstances may have led to a substantially higher percentage of Austrian community pharmacists willing to administer immunisations. However, the current debate does not only raise heated discussions between the medical and pharmacist associations, but also among pharmacists themselves, with some considering immunising as a ‘non-pharmaceutical task’ in the open comments. Our study further demonstrates that Austrian community pharmacists willing to immunise are significantly younger than their counterpart. This can probably be explained by the changing role of pharmacists in the health care system from a drug-focused role to a more patient-facing role [20]. Following examples of the Anglo-American region and political pressure in the last 20 years, Austrian pharmacy schools have included selected clinical pharmacy topics into their undergraduate curricula and have started to offer post-graduate education opportunities to meet the demands of a modern pharmacist [7, 21]. This will have raised the overall awareness and expectations of their professional role profile in younger pharmacists.

In contrast to the high willingness to administer immunisations in general, this study emphasises a reluctance towards pharmacist-provided vaccination of vulnerable groups, i.e. children under 14 years (15.6% willingness, *n* = 49) and adolescents from 14 to 17 years (55.4% willingness, *n* = 174). These findings do not correspond to results from Marra, Kaczorowski and Marra (2010), who conducted a survey among staff pharmacists and pharmacy managers/owners in British Columbia, Canada [22]. They reported that 46.3% (*n* = 57) of respondents were willing to administer vaccines to children under 12 years old. However, the study does not indicate the representativeness of the small sample size and must therefore be interpreted with caution. Whilst safety reasons represent an important barrier to pharmacist-provided immunisation of vulnerable groups, reluctant pharmacists should be made aware of their important role in improving children’s immunisation status. In 1997, Hoeben et al. already emphasised pharmacists’ engagement in childhood immunisations in the USA, where this practice is still widely implemented [23]. Additionally, pharmacists who are generally unwilling to vaccinate showed higher probability to evaluate personnel resources, close patient contact and management of side effects as critical/highly critical. Hence, sufficient staffing and appropriate training to manage side effects, particularly anaphylactic reactions, can be seen as important aspects when attempting to gain further support in pharmacists not willing to immunise. While the probability of anaphylaxis is very low with an incidence of one per 100.000 to one per 1.000.000 doses, it can be life threatening [24]. In line with other countries, where pharmacist-provided vaccination is already implemented, adequate training and standard operating procedures should be in place for Austrian pharmacists to safely manage side effects [25]. In contrast to side effects, acceptance by physicians and financial remuneration were generally rated as the least important requirements for service implementation. This finding may be the result of community pharmacists’ strong attitude towards vaccine administration for the benefit of public health regardless of funding and acceptance which has been sparked by the current national debate and strong Austrian Medical Association opposition [10]. Nevertheless, for a community pharmacy service to be successfully implemented the awareness and acceptance of such an innovation is vital [26]. Pharmacists’ strong attitude towards administration can also be reflected in their rating of the sight of blood and close patient contact as a non-concern. In terms of appropriate education, the majority of participating pharmacists considered the right time for training to be after the foundation training with a 2-yearly renewal, even though continued professional development is not mandatory for Austrian pharmacists up to now. This mirrors recommendations of the General Pharmaceutical Council in Great Britain. They direct the completion of the Declaration of Competence framework, a self-declaration by service-providers that they are service-ready, at least every two years [27]. Regarding appropriate education, participants generally rated practical training more relevant than theoretical topics. The Austrian Chamber of Pharmacists already offered theoretical and practical immunisation training in the beginning of 2021 with the aim of quickly offering a vaccination service in the wake of potential legalisation changes. This had to be suspended due to legal action and political pressure by the Austrian Medical Association. This matches the perception of one pharmacist who commented that ‘physicians and their stakeholders represent an insurmountable hurdle in Austria’ in the open comments. However, after thorough legal review the training could be re-enacted and now represents an important foundation for future legalisation of this service.

This study serves as a valuable instrument for stakeholders attempting to implement a pharmacist provided immunisation-service by highlighting critical requirements and important barriers. For successful service implementation pharmacists themselves should advocate among patients and physicians to obtain sufficient acceptance and emphasise Austrian community pharmacists’ high willingness to administer immunisations.

## Conclusion

This study clearly demonstrates the strong willingness of community pharmacists in Austria to actively administer immunisations in the future. Overcoming critical requirements and barriers, such as sound legislative implementation, adequate liability insurance and appropriate training, will be the basis for successful implementation of this service. Stakeholders should undoubtedly acknowledge pharmacists as highly qualified healthcare professionals in the immunisation process in order to improve patients’ access to immunisations and potentially increase vaccination coverage.

## Data Availability

The datasets analysed during the current study are available from the corresponding author on reasonable request.
